# Hospitals and the Tax-Payer

**Published:** 1920-02-28

**Authors:** 


### The Hospital

The Workers' Newspaper of Administrative Medicine and Institutional Life, Administration, National Insurance and Health.

No. 1760, Vol. LXVII. SATURDAY, FEBRUARY 28, 1920. PRICE TWOPENCE

### HOSPITALS AND THE TAX-PAYER.

The chicken-hearted within the hospital world, and the uninformed without, who desire in the present time of difficulty to jettison the voluntary principle, and attach the hospital system of the country to an already overburdened ministry, should inspect with close attention the figures prepared by the Denison House Committee on Public Assistance. These statistics, which have been supplied to the *Times* by Mr. Geoffrey Drage, the Chairman of the Committee, reveal the enormous increase of expenditure, central and local, arising from various forms of social legislation such as National Health Insurance, Old-age Pensions, Education, Provision of Meals Act, the Poor Law, the Unemployed, and Public Health, which for want of a better description may be described as direct public assistance.

The six million pounds annually required for the expenditure of the voluntary hospitals to-day appear a mere bagatelle when placed side by side with the cost of some of the forms of public spending detailed above; but the point to be chiefly pondered is that once the ball is set rolling, no man can tell where it will stop. In a few years, should the views of the pessimists and the over-hasty "reformers" prevail we may find ourselves with a hospital system, enormously increased in cost, insignificantly increased in efficiency, and probably so altered in character as to be unsuited to its purpose.

Unfortunately, the figures presented are a little out of date, as all statistics prepared with precision on such a scale must inevitably be; they mostly are taken up to the year 1918 and, as one instance, do not include the £10,000,000 extra for old-age pensions hastily voted by Parliament just before Christmas. The return, however, is sufficiently illuminating; it shows that, excluding war pensions, which amount to nearly £21,000,000 for 1918, expenditure under the Acts referred to has risen in round figures from £20,700,000 in 1891 to £37,845,000, in 1901, to £63,147,000 in 1911, and £82,000,000 in 1918 for England and Wales alone. For the whole of the British Isles the totals, subtracting war pensions, show a rise from £25,800,000 in 1891 to £104,000,000 in 1918. The subsidy of £50,000,000 annually required to cheapen the loaf of bread takes no part in the calculations.

One of the most curious and unexpected facts that stands out after an examination of these stupendous figures is that, instead of there being a diminution in the expenditure on the relief of the poor, there is an increase. Medical attention and monetary assistance in sickness are given in return for a minute insurance premium, children are educated free and fed during the days of education, substantial allowances have been forthcoming during unemployment, the aged have received annuities, by a dozen great measures the poor have been helped, and yet the cost of poor relief beyond these forms of assistance now amounts to a total of £19,300,000 a year! While there has been no decrease in Poor-law relief between 1911 and 1918, there has been an increase of £7,000,000 in old-age pensions, an additional expenditure of over £6,000,000 in National Health Insurance, an increase of nearly £3,000,000 in Public Health expenditure, which includes payments for sanatoria, maternity, and child welfare, formerly in some part paid under Poor-law relief.

After making a liberal allowance for the decreased purchasing power of money, a point overlooked by the Denison House Committee, it is clear that the more the long-suffering taxpayer provides the more visionary reformers consider he shall or should provide. It is the duty of those responsible for the fortunes of our voluntary hospital system to bring it home to the taxpayer that a moderate voluntary payment is, apart from the underlying sentiment, probably a less expensive way of helping the hospitals than a payment through the agency of the tax or rate gatherer, and that money spent in this manner will undoubtedly secure better value in return.

